# Exergaming (XBOX Kinect™) versus traditional gym-based exercise for postural control, flow and technology acceptance in healthy adults: a randomised controlled trial

**DOI:** 10.1186/s13102-016-0050-0

**Published:** 2016-08-23

**Authors:** Gillian Barry, Paul van Schaik, Alasdair MacSween, John Dixon, Denis Martin

**Affiliations:** 1 Department of Sport, Exercise and Rehabilitation, Northumbria University, Newcastle upon Tyne, UK; 2 School of Social Science, Business and Law, Teesside University, Middlesbrough, UK; 3 Health and Social Care Institute, Teesside University, Middlesbrough, UK

**Keywords:** Balance, Exergaming, Postural control, Flow state experience, Technology experience, Heart rate

## Abstract

**Background:**

The use of exergaming is a potential alternative to traditional methods of balance training, which can be repetitive and somewhat monotonous. The purpose of this study was to assess the effects of exergaming using XBOX Kinect™ versus traditional gym-based exercise with no virtual stimuli (TGB) on postural control, technology acceptance, flow experience and exercise intensity, in young healthy adults.

**Methods:**

Fifty healthy active adults (age: 33.8 ± 12.7 years, height: 172.9 ± 11.9 cm, weight: 75 ± 15.8 kg) were recruited; 44 completed both baseline and post-intervention data collection. Participants were randomised (blind card) allocation to one of two groups: (1) received balance training using the XBOX Kinect™ and (2) performed traditional gym-based exercise. Exercises were matched for intensity, duration and movement patterns across groups. All participants completed three, 30-minute, exercise sessions a week for four weeks. Postural sway was measured using a Kistler™ Force platform during unipedal standing. Mean heart rate (HR) and rate of perceived exertion (RPE) were collected during each exercise session to determine and verify that intensity of exercise was matched between groups. Technology acceptance was measured with the Unified Theory of Acceptance and Use of Technology (UTAUT) and flow experience with the Flow State Scale (FSS).

**Results:**

Heart rate was matched between groups and BORG RPE was significantly lower in the Kinect™ group. There were significant between-group differences in postural sway in the medial-lateral direction and CoP. There were also significant differences in technology acceptance between groups for performance expectancy, social influence and behavioral intention, with higher values in the Kinect exercise group. The flow state scale showed significant differences between the groups on several dimensions, with higher values in the Kinect exercise group.

**Conclusion:**

Objective physiological demand of exercise (HR) was matched across groups, but the exergaming group perceived it as being less demanding and of lower intensity. This suggests that exergaming may offer an alternative method of rehabilitation exercise through improved concordance. Balance training in healthy adults using the Kinect is both accepted and intrinsically motivating.

**Trial registration:**

Retrospectively registered on 27th July 2016. Trial number NCT02851017.

## Background

Exergaming - exercise with the use of an interactive computer-generated environment - is increasingly used in physical rehabilitation [1, 2]. Benefits have been reported in a range of clinical populations (people with neurological problems [3] children with cerebral palsy [4] and learning difficulties [5], Parkinson’ disease [6], multiple sclerosis [7] and older people [8, 9]). Balance training is an important focus of such rehabilitation. Previous literature regarding the effects of exergaming as a method of balance-training has mainly been conducted using the Nintendo Wii™ and the Wii™ fit where people must stand on a balance board to play the games [10–12]. Results for healthy active older adults suggest the use of the Wii™ fit to be an effective method of balance-training with physiotherapy [13].

In respect to young healthy adult participants, research is limited. Brumels et al. [14] compared four weeks of thrice weekly exergaming with the Dance Revolution™ and Wii™™ systems with traditional balance-based exercise including the Star Excursion Balance Test (SEBT) and mini trampoline. They found significant reductions in anterior posterior postural sway for both Dance Revolution™ (*p* = 0.028) and Wii™™ groups (*p* = 0.043) but no improvement for the traditional exercise group. Yet, the traditional exercise group had significant improvements in SEBT. This may have been because SEBT formed part of the exercises for the traditional exercise group. Vernadakis et al. [15] results concurred with Brumels et al. [14] in that there were significant improvements over time using the Wii™ fit for young adults performing a bi-weekly, 8-week program using the Wii™™ compared to traditional balance-training exercises. However, no significant differences were observed between groups.

Vernadakis et al. [16] also analyzed the effects of the XBOX Kinect™ for ten weeks of bi-weekly balance-training in previously injured young competitive male athletes (*n* = 63). Participants were randomized to one of three groups: 1) exercise with XBOX Kinect™ 2) traditional physiotherapy or 3) no exercise (control group). Dynamic balance was assessed at baseline and after the intervention using the Biodex Stability System, as overall stability index (OSI) and the limits of stability (LOS). Exercise enjoyment was subjectively assessed with the Physical Activity Enjoyment Scale (PACES) at the end of the exercise intervention. Results showed that the two exercise groups improved significantly in overall stability and limits of stability; however, change in the control group was minimal over time and non-significant. The only between-group difference was in the enjoyment rating, where the Kinect™ group showed a significantly higher level of enjoyment over the traditional balance-training group.

Although literature has shown that traditional balance training alone is effective in improving balance in a range of populations [17, 18], studies comparing exergaming with “traditional” balance exercises (SEBT, trampolines and wobble boards) have shown mixed results from both exergaming and traditional balance training groups improving in postural control outcomes [7, 16] to greater improvement in the exergaming group over traditional balance exercise [14, 15]. A potential reason for the differentiation if results could be due to different movements required in the “traditional” balance exercises rather than there being something inherently different about exercising in a virtual environment. There is also a dearth of randomized controlled trials (RCT) in this area so the evidence base is limited.

One important limitation of the Wii™ system, in training balance, is that players are restricted to only moving on the system’s balance board. The XBOX Kinect™ (released in late 2010) was the first commercially available gaming system where truly free movement is possible. The Kinect™ player is free to move their whole body, without restrictions, as neither a balance board, nor a hand-held controller, is required.

Furthermore, few have studied the important psychological aspect of exergaming, in particular acceptance and flow experience. The Unified Theory of Acceptance and Use of Technology (UTAUT) [19] and Flow State Scale (FSS) [20] were used in this study to allow a more in-depth understanding of to what extent people accept and feel immersed in, exergaming. A modified version of the UTAUT has only been applied once to exergaming previously in a group of MS patients [7]. The results from Robinson et al. [7] showed that when comparing exergaming (Wii) to traditional balance exercise there was no significant differences in UTAUT between exercise groups. Yet flow state was significantly greater for those in the Wii exercise group (indicating greater levels of immersion).

The aim of this study was to assess the effects of exergaming using the XBOX Kinect™ system, versus, traditional gym-based exercise, with no virtual stimuli (TGB) on: (1) postural control, (2) technology acceptance (3) flow experience and (4) exercise intensity in young healthy adults. Matching of intensity of exercise, in the two groups, was assessed objectively, by Heart Rate and subjectively by Borg RPE [21] during all exercise sessions. To our knowledge this is the first paper to compare the effects of exergaming against matched traditional exercises where the movement patterns, intensity and physiological demand was matched and assessed across groups.

## Method

### Design overview

A prospective, randomized controlled two-arm trial design: Group 1) exergaming with XBOX Kinect™ and Group 2) traditional gym-based exercise (TGB). All testing was carried out by the first author who was not blind to participant allocation.

### Setting and participants

Ethical approval was granted by Teesside University Research Governance and Ethics Committee. All testing took place at Teesside University physiotherapy laboratory. Convenience sampling was used to recruit active, XBOX Kinect™ naive adult participants. Inclusion criteria: male or female, aged 18–50 years, physically active (three or more moderate-vigorous physical activity sessions per week [22]), free from injury (no musculoskeletal injuries or neurological conditions) and able to take part in four weeks of exercise. Exclusion criteria: unable to give informed consent and/or to comprehend and write English, current (or history of) any medical condition or injury which would contraindicate participation, allergy to alcohol wipes and/or adhesive tape and previous experience of using the XBOX Kinect™.

### Randomization and interventions

After written informed consent, demographic information and baseline outcome data had been collected prior to participants getting randomly allocated to a group by blind card randomization. Both intervention groups were then introduced to the allocated programme, undertaking each of the programme-specific exercises, following which they were asked to complete both the UTAUT and FSS questionnaires. In both groups participants undertook four weeks of allocated exercise, three times per week for 30 min each session. All exercises were completed on a one-to-one basis with the first author supervising the sessions and exercising with the TGB group.

TGB exercises matched the movement patterns and physiological demands of exergaming. The XBOX Kinect™ (Redmond, WA, USA), group played six games (see Table 1 for full description) from Kinect™ Adventures™ (Reflex Ridge and River Rush) and Kinect Sports™ (Boxing). Those in the TGB group performed exercises that were matched for sequence, intensity, duration and mode of exercise by adopting open and closed kinetic chain movements, in the same range and loading as required in the Kinect™ group. For both groups intensity was increased each week staring from the second week by adding 1 kg weights leg weights, increasing to 2 kg leg weights in week 3 and 2 kg leg weight plus 1 kg wrist weights in week 4. The weight loads are relatively light and used to increase energy expenditure, the reason for low weights <50 % of body weight is to familiarise those who are not used to exercising with weights and to allow full body movements [23]. Progression was made in each exercise group depending on proficiency of movement and ability to match the skill level of the game (Kinect group only) and by the first author exercising with the TGB group. Balance data were collected at baseline and after week four (12th session).

**Table 1:** Comparison of movement patterns between exergaming (Kinect) and traditional gym-based (TGB) exercise with no virtual stimuli

Games	Exergaming Instruction	Mirror matched Instruction	Movements required
Reflex Ridge	Steering a cart along a track, avoiding obstacles by jumping and landing on two feet, squatting down, and using full body movements jumping from left to right to avoid barriers and collect points.	Jump up and down on the spot, taking off and landing in the same position (2 footed). In between jumping, perform a squat, keeping your back straight and not bent. Move your full body from left to right when instructed to do so as fast and safely as possible. Only under instruction from GB alternate the movements.	Full medial and lateral weight shifting. Vertical jumping and squatting low.
River Rush	Steering a river raft boat down a rapid to collect points by moving from left to right, reaching out to the sides to grasp points and jumping up and down (taking off and landing on two legs).	Move your whole body from left to right in a fast and safe manor when instructed. On the commend of GB jump, taking off and landing on two feet, either straight up and down or jumping to the left or right. When jumping reach lift both arms up and in the direction of movement (i.e. left or right).	Full medial and lateral weight shifting of the centre of gravity over base of support.
Boxing	Punch and kick as many virtual targets in a specific time (1 min).	Lift both arms up in front of chest and clinch your fingers into a fist position. Using alternative arms punch forward, and punch across your body with twisting of the torso at the same time. For kicking movements kick straight in front at waist height, then alternatively kick across the body, requiring torso twisting.	Anterior and medial-lateral weight shifting of the centre of gravity over base of support. Concentric and eccentric hip and shoulder flexion and extension, with torso twists
Super Saver	Reaching up and forwards and moving legs and torso from side to side to block a ball from going in the goal	Lift both arms up and forwards and grasp your fingers and the drop them back down, move torso from left to right and move legs alternatively to the side.	Full medial and lateral weight shifting of the centre of gravity over base of support. Concentric shoulder flexion, finger flexion and hold and eccentric flexion back to neutral.
Target Kick	Kick virtual ball into the targets as many times as possible, standing on alternative legs to kick the ball.	Start in a normal neutral standing position, (two feet on the ground); alternatively produce kicking movements with each leg.	One legged standing with hip flexion and extension.
Bump Bash	Avoid as many targets thrown over a volleyball net by moving left or right, squatting down or jumping up to avoid the obstacles.	Start in a normal neutral standing alternatively produce squats, followed by jumping and moving to side to side as quickly and safely as possible. Under the instruction of GB the movements were randomised and shouted out to the participants.	Full medial and lateral weight shifting. Vertical jumping and squatting low.

### Outcome measures

The outcome measures were postural sway (Kistler™ force plate), heart rate (HR), technology acceptance (UTAUT questionnaire [19]) and flow experience (FSS questionnaire [20]) recorded at baseline and after the four week intervention.

Postural sway was measured using a portable Kistler™ force platform (Model 9286AA, W 40 × L 60 × H 3.5 cm) with a sampling rate of 1000 Hz [24]. Participants stood barefoot on the Kistler™ force plate, and looked directly ahead at a visual target (black circle) positioned 3 m from the centre of the force plate at eye height [25–27]. Participants were instructed to stand as still as possible with their arms by their side and eyes open [28, 29], on their dominant leg (preferred kicking) for five periods of 30 s. Between trials, participants stepped off the force plate to allow calibration of the equipment which gave a 30 s rest.

Heart rate (HR) was recorded using a Polar™ Heart Rate Monitor™ (FS2C), recording watch and T31 coded chest strap (Polar Electro, Oy, Finland). Mean HR was collected at the end of every exercise session and calculated as a percentage of predicted HR max (220 - age). For a subjective measure of physiological cost the BORG Rate of Perceived Exertion (RPE) scale was used [21]. Mean HR and RPE data were recorded in each exercise session. RPE was defined as how hard participants felt their body was working in general based on the physical sensations they may experience during the activity, including increases in HR, respiration, breathing rate, sweating, and muscle fatigue. The 14 point numerical scale is supported by verbal descriptors, where 6 was defined as “no exertion at all”, 11 “light”, 13 “somewhat hard”, 15 “hard (heavy)”, 17 “very hard” and 20 “maximum exertion”. RPE values between 12 and 13 and 14 and 17 equate to “moderate” and “vigorous” intensity exercise respectively (ACSM, [22]). HR and RPE were taken at 3 separate time intervals during each 30 min session (10, 20 and 30 min) and a mean HR and RPE were calculated of each session.

Technology acceptance was measured using UTAUT which comprised a 7-point Likert scale, with response options on a Likert scale from 1 (strongly disagree) to 7 (strongly agree). The questionnaire has six main domains, performance expectancy (PE; system will help performance), Effort Expectancy (EE; ease of using system), Social Influence (SI; degree in which others believe they should use system), Facilitating Conditions (FC; support in using the system), Self-efficacy (SE; confidence in using the system) and Behavioural Intention (BI; intention to use the system again). The questionnaire was adapted for exergaming and TGB, as previously used in technology settings and validated [14].

Flow state scale (FSS) developed by Jackson and Marsh [20] assessed participants’ level of flow experience into the exercise using exergaming and TGB. Previously this questionnaire has been used in art and science [30, 31], music [32], sport [33, 34], exercise performance [35, 36], gaming [37] and human-computer interaction [38, 39]. The questionnaire consist of a 36-item questionnaire with nine subscales and response options on a Likert scale from 1 (strongly disagree) to 5 (strongly agree). Dimensions of flow include challenge-skill balance (CB; skills match the task and will be successful); clear goals (CG; experience of having a pre-set goal which on is aiming to achieve); unambiguous feedback (UF; feedback on performance); concentration of task (CT; focused on task); paradox of control (PC; performs task with ease); action-awareness-merging (AM; automatic response to task); transformation of time (TT; time speeds up or slows down during activity); loss of self-consciousness (LS; immersed in task) and autotelic experience (AE; activity intrinsically rewarding).

After completing the four-week exercise intervention all participants repeated baseline measures (postural sway, UTAUT and FSS). During all exercise mean HR and RPE were recorded in order to assess physiological cost. All questionnaires were completed unsupervised by the researcher to avoid bias.

### Exergaming system

Exergaming was performed using the Microsoft XBOX Kinect™ (Redmond, WA, USA), this consists of a Kinect sensor (Kinect head - the rectangular part - W110 × D25 × H15 mm) and the base (W30 × D30 × H15 mm) the system does not require any hand-held controller as it relies infrared sensors to detect and track participants’ movements which are used to generate an avatar which is projected in real time into the gaming environment which is displayed on a widescreen plasma screen (37, Hannspree, Type T73B, Greyenstraat 65, Venlo, Netherlands).

### Data extraction

Range and standard deviation of the Centre of Pressure (CoP) displacements in the anterior-posterior (_AP_) and medio-lateral (_ML_) directions (CoP_AP_ SD, CoP_AP_ range, CoP_ML_ SD, CoP_ML_ all mm) and the resultant CoP velocity (mm.sec^−1^) [25, 40] were extracted during unipedal standing assessments using Bioware software (Kistler™) CoP velocity was calculated using previous methods Raymakers [40] after low-pass filtering of the raw data at 10 Hz.

### Statistical analysis

Data were analysed with Statistical Package for the Social Sciences Version 20 for Windows (SPSS™, Chicago, IL, USA). Analysis of covariance (ANCOVA) was conducted on each of the outcome measures, comparing the post-test differences between the groups, with baseline values comprising the covariate. Mixed analysis of variance (ANOVA) was performed to analyse the effect of treatment over time between groups. All analysis used a significance level of 0.05.

## Results

**Fig. 1:**
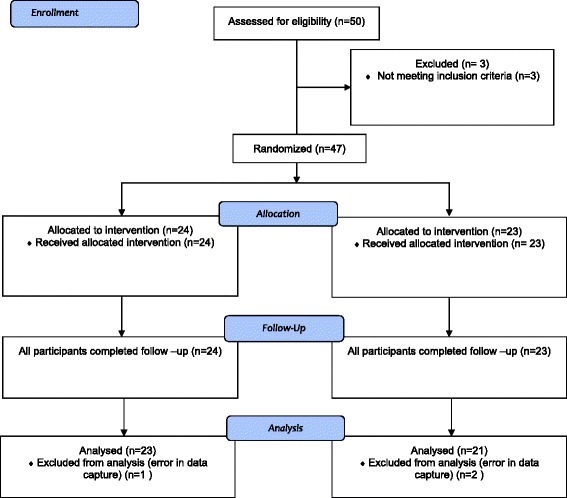
CONSORT flow diagram illustrating a participant entering the study. The final number of participants analysed is based on the principle of complete case analysis and intention-to-treat principle

Fifty healthy participants were screened for eligibility. Three were excluded for not meeting the inclusion criteria (1 amputee and 2 injured). Forty-seven (27 males, 20 females mean age: 33 years, SD 12) were randomly allocated to either the Kinect™ exercise group (*n* = 24) or TGB (*n* = 23), (see Fig. 1 for CONSORT diagram). After post exercise assessment, a further 2 were lost in the TGB group due to technical errors in data capture; therefore, overall 44 participants were analysed at both baseline and after intervention (*n* = 23 Kinect group and *n* = 21 TGB). Descriptive statistics for all outcome measures, before and after intervention, by group, are presented in Table 2.

**Table 2:** Descriptive for outcome measures

	Start of exercise programme		End of exercise programme	
	XBOX Kinect	TGB	XBOX Kinect	TGB
	Mean (SD)	Mean (SD)	Mean (SD)	Mean (SD)
Postural sway				
AP SD (mm)	8.31 (1.44)	7.62 (1.23)	8.27 (1.92)	8.06 (1.45)
AP Range (mm)	45.11 (8.91)	40.83 (7.10)	42.37 (9.50)	41.55 (6.99)
ML SD (mm)	6.12 (1.52)	5.55 (1.40)	5.33 (1.19)	5.51 (0.78)
ML Range (mm)	33.73 (9.06)	31.85 (10.02)	28.31 (5.71)	31.48 (4.44)
COP Velocity (cm.s-1)	55.51 (10.04)	49.64 (10.38)	48.70 (6.96)	46.96 (8.89)
Physiological cost of exercise				
Heart rate	117.56 (17.5)	115.85 (21.85)	150.21 (13.70)	149.57 (6.46)
Rate of perceived exertion (RPE)	9.56 (2.04)	9.29 (1.38)	13.39 (1.44)	14.29 (0.90)
UTAUT				
Performance Expectancy	5.05 (1.12)	5.17 (1.47)	5.93 (1.16)	5.19 (0.94)
Effort Expectancy	5.13 (1.61)	5.38 (1.02)	6.08 (1.12)	5.63 (0.97)
Social influence	5.25 (1.03)	4.88 (1.59)	5.65 (1.21)	4.24 (1.20)
Facilitating Conditions	5.90 (1.39)	5.79 (1.09)	5.88 (1.30)	5.17 (1.32)
Behavioural Intention	5.51 (1.53)	5.50 (1.29)	6.13 (1.26)	4.86 (1.19)
FSS				
Autotelic Experience	3.82 (0.69)	3.36 (0.60)	4.00 (0.910	3.40 (0.55)
Clear Goals	3.87 (0.48)	3.55 (0.53)	4.03 (0.62)	3.56 (0.530
Challenge-Skill Balance	4.14 (0.68)	3.69 (0.58)	4.39 (0.69)	3.83 (0.60)
Concentration of Task	3.75 (0.64)	3.11 (0.70)	3.86 (0.82)	3.10 (0.42)
Paradox of Control	4.03 (0.79)	3.70 (0.62)	4.04 (0.59)	3.61 (0.72)
Unambiguous Feedback	3.83 (0.63)	3.44 (0.65)	4.12 (0.66)	3.53 (0.71)
Action-Awareness Merging	3.70 (0.610	3.43 (0.61)	4.02 (0.77)	3.47 (0.52)
Transformation of Time	3.61 (0.660	2.99 (0.70)	3.91 (0.89)	3.00 (0.84)
Loss of Self-Consciousness	3.97 (0.78)	3.37 (0.60)	4.21 (0.63)	3.65 (0.51)

*Note*. Figures are means, with standard deviations in brackets

### Postural control

An ANCOVA revealed a significant main effect of exercise type for *ML range*, (F [1, 42] = 8.63, *p* = 0.005, ε^2^ = 0.17), in favor of exergaming with better postural control. In an analysis of the effect of treatment over time between groups, a mixed ANOVA showed statistically significant differences in favor of exergaming, in the magnitude of change observed (pre-post intervention) for ML SD (F [1, 44] = 5.77, *p* = 0.02, ε^2^ = 0.12), ML Range (F [1, 44] = 6.15, *p* = 0.02, ε^2^ = 0.13) and CoP velocity (F [1, 44] = 10.47, *p* = 0.002, ε^2^ = 0.20) (see also Table 3). A significant interaction effect between time and exercise was found for ML SD (F [1, 44] = 4.62, *p* = 0.04, ε^2^ = 0.10) in favour of exergaming, as mean (SD) values for ML SD decreased more with a reduction from 6.12 (1.52) mm to 5.33 (1.14) mm than for TGB, from 5.55 (1.40) mm to 5.51 (0.78). A significant interaction effect between time and exercise was also established for ML Range (F [1, 44] = 4.75, *p* = 0.04, ε^2^ = 0.10) also in favour of exergaming, with a larger reduction in ML range over time from 33.67 (9.11) mm to 28.23 (5.74) mm than with TGB 31.85 (10.02) mm to 31.48 (4.44) mm. No other significant differences were established for any of the other postural-control variables.

**Table 3:** Adjusted post-intervention between group difference (ANCOVA) and within-group change over time mean differences (95 % CI) and for outcome measures

	Adjusted post-intervention difference between groups (ANCOVA)	Within-group change over time (Mixed ANOVA)	
Outcome	XBOX™ - TGB	XBOX™	Traditional gym based (TGB)- Exercise
AP SD	−0.20 (−1.12 to 0.78)	0.50 (−0.62 to 0.72)	−0.45 (−1.24 to 0.34)
AP Range	−1.77 (−6.13 to 2.59)	2.74 (−0.39 to 5.86)	0.72 (−4.30 to 2.85)
ML SD	−0.42 (−0.93 to 0.80)	0.79 (0.36 to 1.22)*	0.04 (−0.55 to 0.63)
ML Range	−3.83 (−6.41 to −1.23)**	5.44 (2.78 to 8.10)*	0.37 (−3.78 to 4.52)
COP Velocity	−0.32 (−4.88 to 4.24)	6.81 (1.93 to 11.70)**	2.67 (−0.78 to 6.13)
Performance Expectancy (PE)	0.78 (0.19 to 1.38)*	−0.89 (−1.33 to −0.43)*	−0.02 (−0.72 to 0.67)
Effort Expectancy (EE)	0.53 (−0.70 to 1.12)	−0.94 (−1.52 to −0.37)**	−0.25 (−0.86 to 0.36)
Social influence (SI)	1.32 (0.59 to 2.05)**	−0.40 (−0.94 to 0.13) **	0.64 (−0.10 to 1.39)
Facilitating Conditions (FC)	0.67 (−0.08 to 1.42)	0.01 (−0.33 to 0.36)	0.62 (−0.25 to 1.48)
Behavioural Intention (BI)	1.36 (0.65 to 2.01)***	0.88 (−1.42 to −0.35)***	0.65 (0.14 to 1.45)
Autotelic Experience	0.35 (−0.09 to 0.79)	−0.18 (−0.46 to 0.09)	−0.05 (−0.42 to 0.33)
Clear Goals	0.34 (−0.01 to 0.69)	−0.16 (−0.36 to 0.03)	−0.12 (−0.35 to 0.33)
Challenge-Skill Balance	0.33 (−0.04 to 0.70)	−0.25 (−0.44 to −0.57)*	−0.14 (−0.51 to 0.23)
Concentration of Task	0.45 (0.05 to 0.85)*	−0.11 (−0.34 to 0.11)	−0.00 (−0.36 to 0.36)
Paradox of Control	0.52 (0.10 to 0.94)*	−0.21 (−0.53 to 0.120	0.07 (−0.35 to 0.31)
Unambiguous Feedback	0.33 (−0.06 to 0.73)*	−0.23 (−0.46 to −0.00)	−0.11 (−0.52 to 0.31)
Action-Awareness Merging	0.44 (0.05 to 0.82)*	−0.33 (−0.54 to −0.11)	−0.05 (−0.45 to 0.35)
Transformation of Time	0.60 (0.06 to 1.14)*	−0.30 (−0.55 to 0.06)	−0.01 (−0.51 to 0.49)
Loss of Self-Consciousness	0.38 (0.007 to 0.75)*	−0.22 (−0.50 to 0.50)*	−0.32 (−0.07 to 0.70)
HR	0.37 (−6.05 to 6.79)	−32.65 (−39.79 to −25.51)***	−33.71 (−43.89 to −25.59)***
RPE	−1.01 (−1.60 to −0.45)***	−3.83 (−4.46 to −3.20)***	−5.00 (−5.58 to −4.42)***

*p < 0.05. **p < 0.01. ***p < 0.001

### Physiological cost of exercise

In order to assess success of matching exercise intensity, across groups, mean HR and RPE was compared. ANCOVA showed no statistically significant post-intervention differences for HR, indicating that intensity of the exercise was matched between groups. Despite no difference in mean HR, there was a statistically significant difference between the groups for mean RPE (F [1, 44] = 12.30, *p* = 0.001, ε^2^ = 0.23). The Kinect™ group perceived less physical exertion than the TGB group. Mixed ANOVA showed that over time there was a significant increase in mean HR (F [1, 44] = 126.97, *p* < 0.001, ε^2^ = 0.75) in both exercise groups (Kinect and TGB). This would be expected owing to increase in intensity of the exercise and therefore elicit physiological responses to exercise intensity. The same occurred in relation to RPE: over time this significantly increased, (F [1, 44] = 452.9, *p* < 0.001, ε^2^ = 0.91) in both exercise groups (See Table 3).

#### UTAUT

Overall, UTAUT scores were higher after the intervention in the Kinect™ group than in the TGB group (See Table 3). ANCOVA showed statistically significant post-intervention differences between groups for performance expectancy (PE) (F [1, 44] = 6.99, *p* = 0.012, ε^2^ = 0.15); social influence (SI) (F [1, 44] = 13.35, *p* = 0.001, ε^2^ = 0.25); and behavioral intention (BI) (F [1, 44] = 14.91, *p* < 0.001, ε^2^ = 0.27). Higher mean values occurred in the Kinect™ group, indicating a greater level of acceptance towards exergaming rather than TGB group.

Mixed ANOVA showed that over time there was a significant increase in performance expectancy (PE), (F [1, 44] = 5.35, *p* = 0.03, ε^2^ = 0.11), and effort expectancy (EE), (F [1, 44] = 8.83, *p* < 0.01, ε^2^ = 0.17). Statistical significant differences were also found for and interaction effect of time and exercise for social influence (F [1, 44] = 5.76, *p* = 0.02, ε^2^ = 0.12), and behavioral intention (F [1, 44] = 11.52, *p* < 0.001, ε^2^ = 0.21). For both variables, the Kinect™ exercise group showed higher mean values after intervention for SI and BI, whereas in the TGB group there was a reduction in mean scores. No statistically significant effects were established for the remaining UTAUT subscales.

#### Flow state scale

Overall, the scores on the flow subscales were higher at both baseline and after exercise in the Kinect™ group than in the TBG group (Table 2). ANCOVA showed significant differences between groups for concentration of task (F [1, 44] = 5.16, *p* = 0.03, ε^2^ = 0.11), paradox of control (F [1, 44] = 5,16, *p* = 0.03, ε^2^ = 0.11), feedback (F [1, 44] = 4.43, *p* = 0.04, ε^2^ = 0.10), action-awareness merging, (F [1, 44] = 5.21, *p* = 0.03, ε^2^ = 0.11), transformation of time (F [1, 44] = 5.02, *p* = 0.03, ε^2^ = 0.11), and loss of self-consciousness, (F [1, 44] = 4.23, *p* = 0.05, ε^2^ = 0.09). No significant effects were observed for the remaining variables. No significant time-by-exercise type interaction effect was found for any of the flow variables.

## Discussion

The aim of this study was to assess the effects of exercise type (exergaming using the XBOX Kinect™ system or traditional gym-based exercise, with no virtual stimuli (TGB) on (1) postural control, (2) technology acceptance (3) flow experience and (4) exercise intensity in young healthy adults. This is the first study to directly compare exergaming versus traditional gym-based exercise (TGB) with matched exercise in both groups. HR was matched between exercise groups, showing that exercise intensity was well matched between the Kinect™ and TGB; in both participant’s experienced moderate-to-vigorous levels of exercise intensity (between 61 and 82 % HR max). Overall, we found postural control could be improved by using the Kinect™ as well as high levels of technology acceptance and flow.

### Postural control

We observed significant reductions in sway in CoP_ML_ SD, CoP_ML_ range, and CoPv, all in favour of the Kinect™, indicating better postural control. In relation to between-group differences, there was a significant difference between groups for CoP_ML_ range, again with greater reductions for the Kinect™ exercise group. Moreover, the statistically significant differences between the exercise groups were substantial (effect size: 0.10 < ε^2^ < 0.20). While these differences were statistically significant, it is unclear to the authors what level of improvement would be clinically meaningful, and so inferences about clinical relevance should be made with caution. This support earlier work from Vernadakis et al. [16] who found in previosuly injured young males that Kinect™ improved postural stability over a 10-week training program. It should be noted, however, that their physiotherapy group also improved over time and the only improvement between groups occurred between both the physiotherapy group and Kinect™ group, and the control group with no exercise. These results though are encourgaing for the use of the Kinect™ in balance-training and would indicate the potential for use of the Kinect™ for balance-training in young healthy and older adults.

In relation to young healthy adults our results also reflect those of Brumels et al. [14] who found significant improvements in postural control in favour of exergaming (Wii™ and Dance Dance Revolution) compared to traditional exercise. Again, it should be noted that their traditional exercise group did significantly improve on the star excursion balance test (SEBT) compared to their exergaming groups; this, however, may have been a learning effect as their traditional exercise group practiced the SEBT during their exercise intervention. It is encouraging that our results are comparable with other literature that found a greater improvement in the exergaiming group over traditional or TGB exercise as a promising new method of training postural control using interactive technology. A possible reason why we found improvements in the ML direction between exercise groups could be the immersive and purposeful nature of the exergaming compared to TGB exercise: while we aimed to match the movements in both groups, it may be that the exergaming group used more rapid movements and movements outwith the base of support to control the avatar moving in a medial-lateral direction.

To our knowledge this is the first study to report a comparison of exergaming with a demonstrably matched exercise programme and hence, isolated, with more confidence, any effects of exergaming.

### Physiological cost of exercise

Both exercise groups produced moderate-vigorous levels of physical activity in accordance with the American College of Sports Medicine Guidelines (ACSM [22]) - moderate intensity exercise HR should be 64–77 % of max HR, and vigorous (hard) intensity 77–94 % max HR. Mean HR for our Kinect™ exercise group ranged from 61 to 82 % across the four weeks of exercise and the TGB ranged from 61 to 78 % of HR max. These results broadly concur with O’Donovan and Hussey [41] who found that playing the Nintendo Wii™ in young healthy adults that Wii™ fit Jogging elicited 71 % of max HR, yet Wii™ boxing, baseball and tennis achieved less than 60 % max HR. These results suggest that games requiring more dynamic movement (jogging and Kinect games) demand greater physiological response and energy expenditure. This also reflects Barry et al. [42] who found that young males achieved moderate intensity exercise [64–72 % HRmax] for exergaming using the XBOX Kinect™. The results indicate that moderate intensity exercise is achievable using the XBOX Kinect in young healthy adults.

No significant difference was found between groups for mean HR, yet, RPE level was significantly and substantially lower in the exergaming group (effect size ε^2^ = 0.23). This reduced perception of effort could well be attributed to the immersive nature of exergaming. Vernadakis et al. [16] supported the notion that people playing the Kinect™ experienced a greater level of enjoyment than traditional physiotherapy, which would support that explanation. Similarly, in a study of the Nintendo Wii™, Brumles et al. [14] reported that those in the Wii™ and DDR group perceived the exercise to be less streneous and more enjoyable than traditional exercise.

### UTAUT

This was the first study to apply a modified version of the UTAUT accross exergaming and TGB. The results showed that, in comparison to TGB, social influence, performance expectancy and behavioural intention were significantly higher in the exergaming group. In essence, particpants exergaming believed that the exercises they were doing would improve their balance (performance expectancy) and they would continue to do those exercises (behavioural intention) and that their peers would encourage this method of exercise (social influence) more so than those doing TGB. These results are encouraging as there is a distinct lack of research regarding acceptance of exergames as a means of exercise. Only one other study to date, Robinson et al. [7] applied the UTAUT in a group of people with MS using the Nintendo Wii™ and standard balance exercise. Their results showed that measures of performance expectancy and effort expectancy were higher in the Nintendo™ group.

### Flow state scale

In the current study we found significantly higher scores in the exergaming group compared to the TGB in six of the nine subscales post-intervention. This indicates that those in the exergaming group were more focused on what they had to achieve (concentrated on task), felt at ease in playing the game (paradox of control), had clear feedback during the game (unambiguous feedback), movements appeared to be automatic (action-awareness merging), and altered their perception of time (transformation of time) and felt immersed in the activity (exergaming, (loss of self-consciousness). Robinson et al. [7] found similar results when appling FSS to exergaming in a group of people with MS using the Nintendo Wii and traditional balance exercise. The results concurred with our study findings, in which five of the nine subscales were significantly higher in the Wii group. A potential reason for these similar findings could be the immersive nature of exergaming when skill sets of the player match the game [37] and the distraction element that is associated with gaming [43].

### Limitations of study

Although this study adopted a classic RCT design (which is unusual within the published literature on exergaming) there were limitations to the study. The sample size is relatively small and a larger study employing multi-centre recruitment is merited. For logistical reasons the primary researcher had to both deliver the interventions and collect the outcome data. We acknowledge this opens the possibility of bias but the use of objective outcome measures offsets that. Interestingly, the control findings showed no improvement in balance in any of the parameters. This highlights a limitation of the transferability of the findings to clinical situations. The control group exercises had a degree of clinical artificiality - they would not be used directly as a clinical programme to improve balance. They were designed specifically, and served that purpose, as an experimental control for the exergaming exercises to provide insight into the effects of the immersive aspects of exergaming. A challenge for future work is to design a study that provides a fair comparison between clinically valid balance exercise and a similar programme carried out within an exergaming environment. We would also propose that care be taken translating the results of supervised lab based exercise to clinical practice where a community, self-directed approach is preferred. As a result we would recommend future work examining home-based exergaming and more RCT designs with larger sample sizes.

## Conclusions

Our results suggest that exergaming, using the XBOX Kinect™, has the potential to enhance postural control compared to standard exercise, a technology for rehabilitation exercise that meets with users’ acceptance and encourages a positive experience of exercise. Exergaming can invoke moderate levels of physical exercise intensity with positive feelings about exercise and reduced perception of effort, over traditional exercise modes. As a result, it may aid concordance with physical rehabilitation regimens. These findings are encouraging for health professionals who may wish to use exergaming for balance training and broader rehabilitation aims, for clinical populations, as it has been shown to be a well-received method of exercise.

## Abbreviations

AP: Anterior-posterior
BORG RPE: Borg Rate of Perceived Exertion
CoP: Centre of pressure
DDR: Dance Dance Revolution
FSS: Flow state scale
HR: Heart rate
LOS: Limits of stability
ML: Medial-lateral
OSI: Overall stability index
PACES: Exercise enjoyment was subjectively assessed with the Physical Activity Enjoyment Scale
SEBT: Star excursion balance test
TGB: Traditional gym-based exercise with no virtual stimuli
UTAUT: The Unified Theory of Acceptance and Use of Technology
